# Recent Advances in Functional and Modified Sponge Materials for Antimicrobial Applications

**DOI:** 10.1021/acsmaterialsau.6c00142

**Published:** 2026-09-03

**Authors:** Adam Brooks Goodman, Elizabeth J. Brisbois

**Affiliations:** School of Chemical, Materials, and Biomedical Engineering, College of Engineering, 1355 University of Georgia, Athens, Georgia 30602, United States

**Keywords:** sponge, antimicrobial, natural polymer, synthetic polymer, biomaterial, infection prevention, biomedical, environmental, food safety

## Abstract

Microbial contamination remains a persistent challenge across healthcare, environmental, and food production sectors, contributing to delayed wound healing, increased healthcare costs, and the spread of water- and foodborne diseases. The growing prevalence of antimicrobial resistance further highlights the need for alternative strategies to effectively control pathogenic microorganisms. Antimicrobial sponges have emerged as promising platforms because of their high porosity, large surface area, and ability to incorporate a wide range of antimicrobial agents. These materials can be constructed from natural and synthetic polymers as well as hybrid matrices, enabling the integration of disinfectants, essential oils, metal-based agents, and other bioactive compounds to enhance antimicrobial performance. This Review summarizes recent advances in the design and development of antimicrobial sponge materials, with particular emphasis on how material composition and antimicrobial strategies influence performance across different applications. Applications across biomedical, dental, environmental, and food-related fields are discussed to highlight the versatility of the sponge-based antimicrobial platforms. Finally, current challenges and future directions aimed at improving the performance, functionality, and translational potential are outlined.

## Graphical abstract

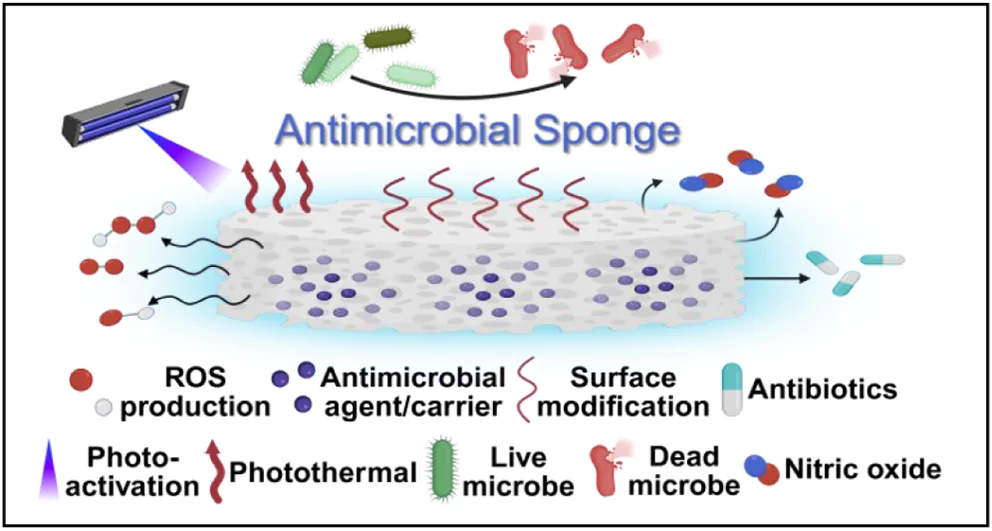

## 1 Introduction

Antimicrobial materials have become essential tools in addressing the growing threat of microbial contamination across healthcare, environmental, and food systems. The rise of drug-resistant pathogens, along with the widespread formation of biofilms in both clinical and nonclinical settings, has intensified the demand for effective strategies to control harmful microorganisms. Traditional antimicrobial approaches, including antibiotic therapy and disinfectants, remain valuable but often suffer from limitations, such as reduced efficacy against biofilms and the increasing prevalence of antimicrobial resistance.^1,2^

Among emerging material-based strategies, antimicrobial sponges and porous matrices have attracted significant attention because of their high porosity, large surface area, and the ability to incorporate a wide range of antimicrobial agents. These structural characteristics enable efficient microbial contact, high loading capacity for antimicrobial compounds, and controlled release behavior, making sponge materials promising platforms for preventing microbial colonization and infection. As a result, antimicrobial sponge materials are increasingly being explored for applications ranging from wound dressings and medical device coatings to water purification and food preservation systems (Figure 1).

**Figure 1:**
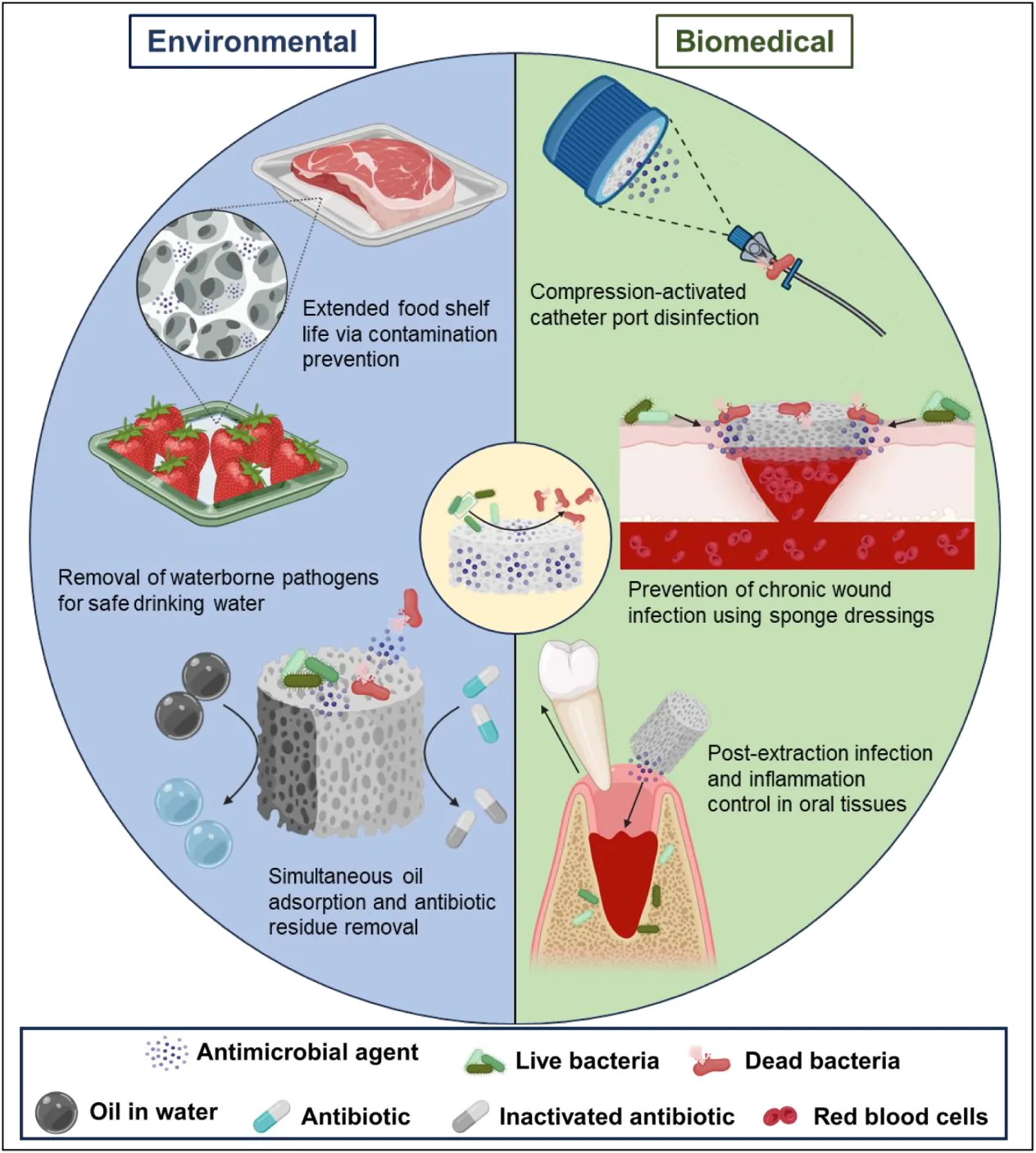
Environmental and biomedical applications of antimicrobial sponges encompass food preservation, water purification, antibiotic neutralization, disinfection, wound healing, and dentistry. The figure was generated using BioRender.com software.

Microbial contamination remains a persistent challenge in healthcare environments, particularly in association with medical devices and chronic wounds. Medical devices such as catheters, endotracheal tubes, and pacemakers are highly susceptible to microbial adhesion and subsequent biofilm formation, which can lead to difficult-to-treat infections.^3^ Healthcare-associated infections affect approximately 1.7 million hospitalized patients annually in the United States and contribute to nearly 100,000 deaths each year.^4^ Biofilm formation further complicates treatment by limiting antibiotic penetration and promoting antimicrobial resistance.^5^

Chronic and acute wounds also present significant risks of microbial infection. Bacterial contamination can delay healing by damaging tissue cells, disrupting blood supply, and promoting biofilm formation.^6^ Surgical site infections represent approximately 14–17% of hospital-acquired infections and contribute substantially to healthcare costs.^7,8^ In addition, diabetic foot ulcers affect a large proportion of individuals with diabetes, with infections frequently leading to severe complications such as limb amputation.^9^

Beyond healthcare settings, microbial contamination also poses major risks to water systems and food supply chains. Pathogenic microorganisms such as *Escherichia coli* (*E. coli*), *Salmonella enterica* (*S. enterica*), and *Vibrio cholerae* (*V. cholerae*) frequently contaminate drinking water and contribute to widespread waterborne diseases.^10,11^ Similarly, microbial contamination during food harvesting, processing, and storage can result in large-scale foodborne outbreaks and significant public-health consequences.^12^

In response to these challenges, antimicrobial sponge materials have emerged as versatile platforms that integrate antimicrobial agents while maintaining structural properties that are suitable for biomedical and environmental applications. Their highly porous architecture enables efficient loading of antimicrobial compounds and promotes diffusion-mediated microbial inactivation, positioning sponge materials as promising candidates for next-generation antimicrobial technology.

## 2 Design Rationale for Antimicrobial Sponge Materials

Antimicrobial sponge materials have gained attention because their unique structural and chemical design features enable effective microbial control across biomedical and environmental applications. The performance of sponge-based systems is strongly influenced by polymer chemistry, surface functionalization, and porous architecture, which collectively regulate antimicrobial loading, diffusion behavior, and material-microbe interactions.

The chemical composition of the polymer backbone plays a critical role in determining material properties (Figure 2). Functional groups such as hydroxyl, amine, and carboxyl moieties can enhance hydrophilicity, promote hydrogen bonding, and facilitate ionic interactions, which influence the swelling behavior, adsorption capacity, and antimicrobial agent incorporation. Hydrophobic segments within polymer matrices can improve mechanical stability and modulate release kinetics, demonstrating that backbone chemistry directly contributes to performance optimization in sponge materials.

**Figure 2:**
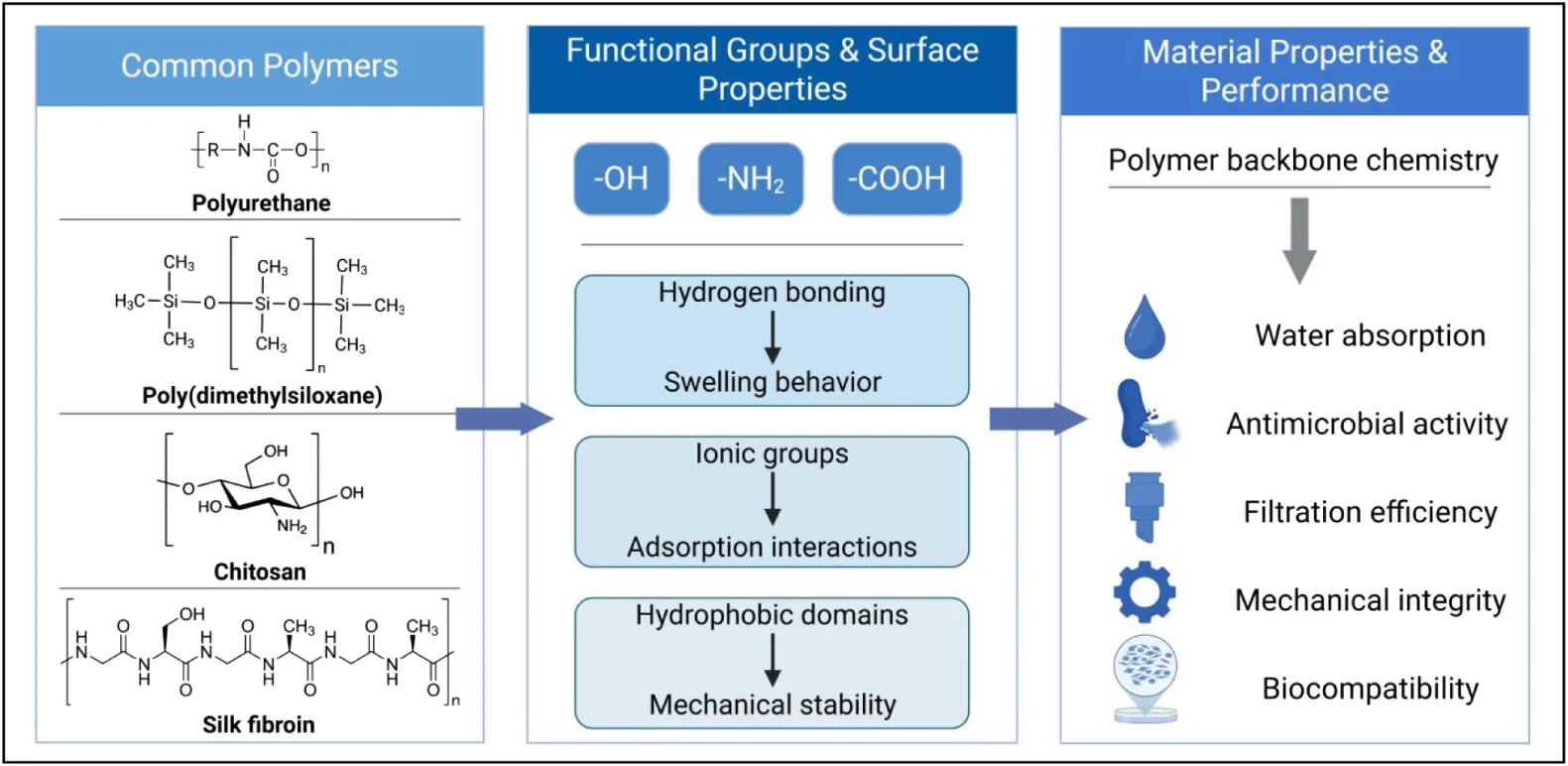
Influence of polymer chemistry on the structural and functional properties of sponge materials. Functional groups and the backbone composition of common polymers regulate hydrophilicity, swelling behavior, antimicrobial agent loading, and diffusion-mediated release, ultimately determining performance in absorption, filtration, and biomedical applications. The figure was generated using BioRender.com software.

The structural characteristics of sponge materials further distinguish them from those of conventional antimicrobial surfaces. The three-dimensional porous network provides a high surface area for antimicrobial agent loading and promotes efficient microbial contact and diffusion-mediated transport of active compounds. Compared with flat materials such as gauze, sponge structures can retain and gradually release antimicrobial agents more effectively, enabling improved antimicrobial performance.^13^ Pore size, porosity, and interconnectivity can be tailored during fabrication to achieve either rapid microbial inactivation or sustained antimicrobial release profiles, depending on application requirements. Recent advances in sponge material design have focused on optimizing architecture and composition through techniques such as freeze-drying, electrospinning, porogen templating, and additive manufacturing, allowing for precise control over antimicrobial distribution and release behavior.^14–17^

Material flexibility and compressibility are additional advantages of sponge systems. These mechanical properties allow sponge materials to conform to irregular surfaces, including wound sites and medical device interfaces, thereby enhancing contact with contaminated environments. Such conformability is particularly beneficial for applications involving catheter access ports and surgical wound management, where localized antimicrobial delivery is required.^18^ Beyond biomedical applications, sponge materials demonstrate a high liquid absorption capacity, enabling the retention of water or organic fluids and extending contact time between antimicrobial agents and microbial contaminants. This property supports applications in water purification, oil absorption, and sustainable food-packaging systems.^19^

Integrating polymer chemistry, porous architecture, and tunable fabrication strategies enables the rational design of antimicrobial sponges for diverse applications. The following sections review the major classes of antimicrobial agents incorporated into sponge matrices and the strategies used to enhance their antimicrobial activity.

## 3 Antimicrobial Sponges: Innovations and Applications

### 3.1 Sponges Fabricated from Polymers with Intrinsic Antimicrobial Properties

To expand the functional utility of sponges, antimicrobial agents are often incorporated into their matrices to impart microbicidal activity. In some cases, however, the sponge material itself possesses inherent antimicrobial properties because of its chemical composition. Sponges fabricated from such materials provide an intrinsic antimicrobial functionality that can be further enhanced through additional agents.

Chitosan is one of the most widely used materials for antimicrobial sponge fabrication. Derived from the deacetylation of chitin obtained from crustaceans, chitosan is valued for its biodegradability, biocompatibility, and low toxicity.^20^ Its antimicrobial activity arises through several mechanisms, including electrostatic interactions with negatively charged microbial cell membranes, chelation of essential metal ions, and interference with DNA/RNA and protein synthesis.^21^ Differences in bacterial cell wall structures influence these interactions, with Gram-negative bacteria possessing a thinner peptidoglycan layer and an outer lipopolysaccharide membrane that can affect susceptibility.^22^ Chitosan also exhibits antifungal activity through interactions with fungal cell walls and membranes, with efficacy influenced by factors such as molecular weight, degree of deacetylation, and environmental pH.^21^

Chitosan sponges are commonly fabricated via freeze-drying. Chitosan is typically dissolved in dilute acetic acid, crosslinked with agents such as calcium,^23^ glutaraldehyde,^24^ or natural acids such as lemon juice,^25^ and cast into molds before freezing and lyophilization. Ice removal during freeze-drying generates a porous, crosslinked network that forms the sponge structure. These materials demonstrate measurable antibacterial activity (Figure 3A), often showing stronger inhibition of Gram-positive bacteria than Gram-negative strains,^26^ although similar effects against both groups have also been reported.^27,28^ Chitosan sponges have therefore been widely explored for applications including microbial removal from contaminated water and infection prevention in wounds such as traumatic, diabetic, and surgical injuries.^15,29^

**Figure 3:**
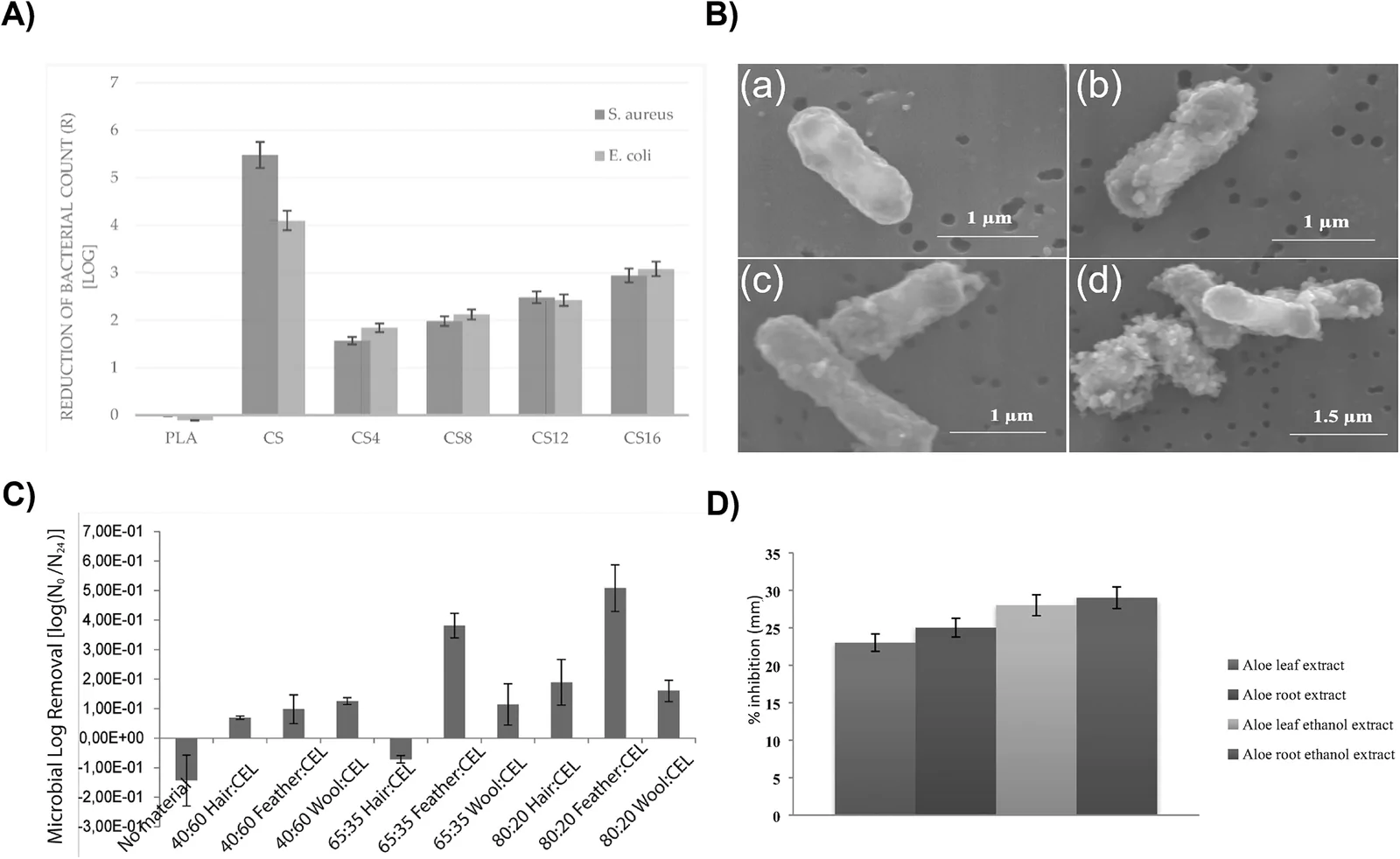
Representative antimicrobial performance of intrinsically antimicrobial sponge-based materials against common microbial strains. **A)** Effect of increasing chitosan (CS) content in CS/polylactic acid (PLA) sponges on antibacterial activity against *E. coli* and *S. aureus*,^26^ reprinted with permission under a Creative Commons CC-BY 4.0 license from [26]. Copyright 2019 MDPI. **B)** Scanning electron microscopy images of *E. coli* before (a) and after (b–d) treatment with an electrochemical graphene oxide (GO)-based sponge,^36^ reproduced with permission from Elsevier, Copyright © 2023. **C)** Antibacterial activity of keratin-derived proteins incorporated into cellulose (CEL) matrices against methicillin-resistant *S. aureus* (MRSA),^38^ reproduced with permission from Elsevier, Copyright © 2016. **D)** Inhibition of *S. aureus* growth by aloe vera-derived antimicrobial components, reprinted with permission under a Creative Commons CC-BY 4.0 license from ref 39. Copyright 2021 John Wiley & Sons, Ltd.

Graphene oxide (GO), an oxidized derivative of graphene containing epoxide, hydroxyl, carbonyl, and carboxyl functional groups, has also been used to fabricate intrinsically antimicrobial sponges.^30^ The antimicrobial activity of GO is generally attributed to direct physical interactions with the bacterial membranes. These interactions can lead to membrane disruption, leakage of intracellular components, and, eventually, cell death. Additionally, GO sheets can envelop bacterial cells, restricting nutrient transport and inhibiting proliferation.^31^ As with many antimicrobial materials, differences in bacterial cell wall structures influence susceptibility, with Gram-positive bacteria often exhibiting greater sensitivity because of the absence of an outer membrane barrier.^32^

Graphene oxide sponges can be fabricated through freeze-drying,^33^ chemical reduction-assisted self-assembly using templates such as vitamin C,^34^ or hydrothermal methods in which GO-coated substrates are treated in an autoclave to generate porous structures.^35^ Beyond their antimicrobial activity, GO sponges possess conductive and electrochemical properties due to their conjugated carbon network and oxygen-containing functional groups, enabling applications in electrochemical sensing, energy storage, and electrically assisted water purification systems, where microbial contaminants can be adsorbed and inactivated. These characteristics have also been exploited for the removal of microorganisms such as *E. coli* from contaminated water (Figure 3B).^36^ In biomedical contexts, GO sponges have further been explored for wound healing and hemostatic applications, where their antimicrobial activity helps prevent infection at the wound site.^37^

Keratin, a fibrous structural protein present in hair, nails, feathers, and skin, has also been investigated as a sponge-forming biomaterial. Its mechanical strength and stability arise from disulfide bonds formed by cysteine residues, making it useful in biomaterial design.^38^ Although keratin exhibits relatively modest antimicrobial activity, its efficacy can depend on protein source, structure, and concentration.^40,41^ Structural variations, such as lower α-helix content and more disordered conformations, have been associated with enhanced antimicrobial activity, particularly against methicillin-resistant *Staphylococcus aureus* (MRSA) (Figure 3C).^38^ Keratin with higher α-helix content has also shown limited activity against *Staphylococcus aureus* (*S. aureus*) and *E. coli*.^42^ Keratin-based sponges fabricated by freeze-drying have therefore been explored primarily for wound healing and tissue engineering applications, where inherent bioactivity and compatibility with biological tissues are essential.^43^

Natural plant-derived materials can also contribute intrinsic antimicrobial activity. Aloe vera (AV) gel, obtained from the leaves of the tropical Aloe vera plant, contains a complex mixture of polysaccharides, vitamins, minerals, proteins, lipids, and bioactive compounds.^44^ Among these components, anthraquinones and salicylic acid derivatives contribute to the plant’s antimicrobial, anti-inflammatory, and wound-healing properties.^45^ Extracts of AV have demonstrated antimicrobial activity against pathogens such as *S. aureus* (Figure 3D) and drug-resistant bacteria by inhibiting biofilm formation and disrupting microbial cellular processes.^39,46^ Freeze-drying of AV gel produces porous sponge-like structures suitable for biomedical applications, particularly wound healing and regenerative medicine.^47^ Aloe vera-derived materials have also demonstrated antifungal activity, including inhibition of *Candida albicans* (*C. albicans*) growth in zone-of-inhibition assays.^48^

Although these intrinsically antimicrobial polymers exhibit measurable antibacterial and, in some cases, antifungal activity, the level of antimicrobial efficacy required ultimately depends on the intended application. For applications such as wound healing, food preservation, and water treatment, reducing the microbial burden may be sufficient to delay contamination, support tissue repair, or improve product stability.^49^ In contrast, indwelling medical devices require high antimicrobial efficacy to prevent initial bacterial adhesion and biofilm formation. Studies reporting reductions approaching or exceeding 4 logs are generally considered particularly promising for these applications,^50^ although the clinically required level of bacterial reduction depends on factors including device type, implantation site, and pathogen. Therefore, intrinsic antimicrobial activity alone is often insufficient for these high-risk applications, motivating the incorporation of additional antimicrobial agents or functionalization strategies to further enhance the antimicrobial performance.

### 3.2 Enhancing Intrinsically Antimicrobial Sponges with Additional Agents

Incorporating antimicrobial agents, including metals, essential oils, and antimicrobial peptides, provides a versatile strategy for enhancing the performance of intrinsically antimicrobial sponges. As alternatives to traditional antibiotics, these agents can inhibit microbial growth and biofilm formation through different mechanisms. When combined with inherently microbicidal matrices, this approach enables synergistic antimicrobial activity, improving bacterial decontamination in environmental applications and reducing the infection risk in wound healing by limiting microbial adhesion and proliferation.

Chitosan-based sponges remain among the most widely used platforms in both biomedical and environmental applications because of their intrinsic biocompatibility, biodegradability, and cationic nature. The amino groups of chitosan can form ionic linkages with carboxyl-containing biopolymers such as gelatin and alginate, enabling the formation of composite networks with tunable properties (Figure 4A).^51^ This ability to form composite systems plays a critical role in modulating antimicrobial performance, particularly when combined with loaded agents, as chitosan is frequently integrated with both natural and synthetic polymers to tailor mechanical strength, stability, and antimicrobial behavior for specific applications.^52–60^ The selection of co-polymers plays a critical role in modulating antimicrobial performance, particularly when combined with loaded agents. For example, chitosan sponges crosslinked with gelatin, alginate, or collagen and loaded with equal amounts of curcumin exhibited markedly different behaviors depending on the secondary polymer.^61^ Among these, gelatin-chitosan sponges demonstrated enhanced curcumin release, resulting in stronger antibacterial activity with inhibition zones of 24 mm and 18 mm against *E. coli* and *S. aureus*, respectively (Figure 4B).

**Figure 4:**
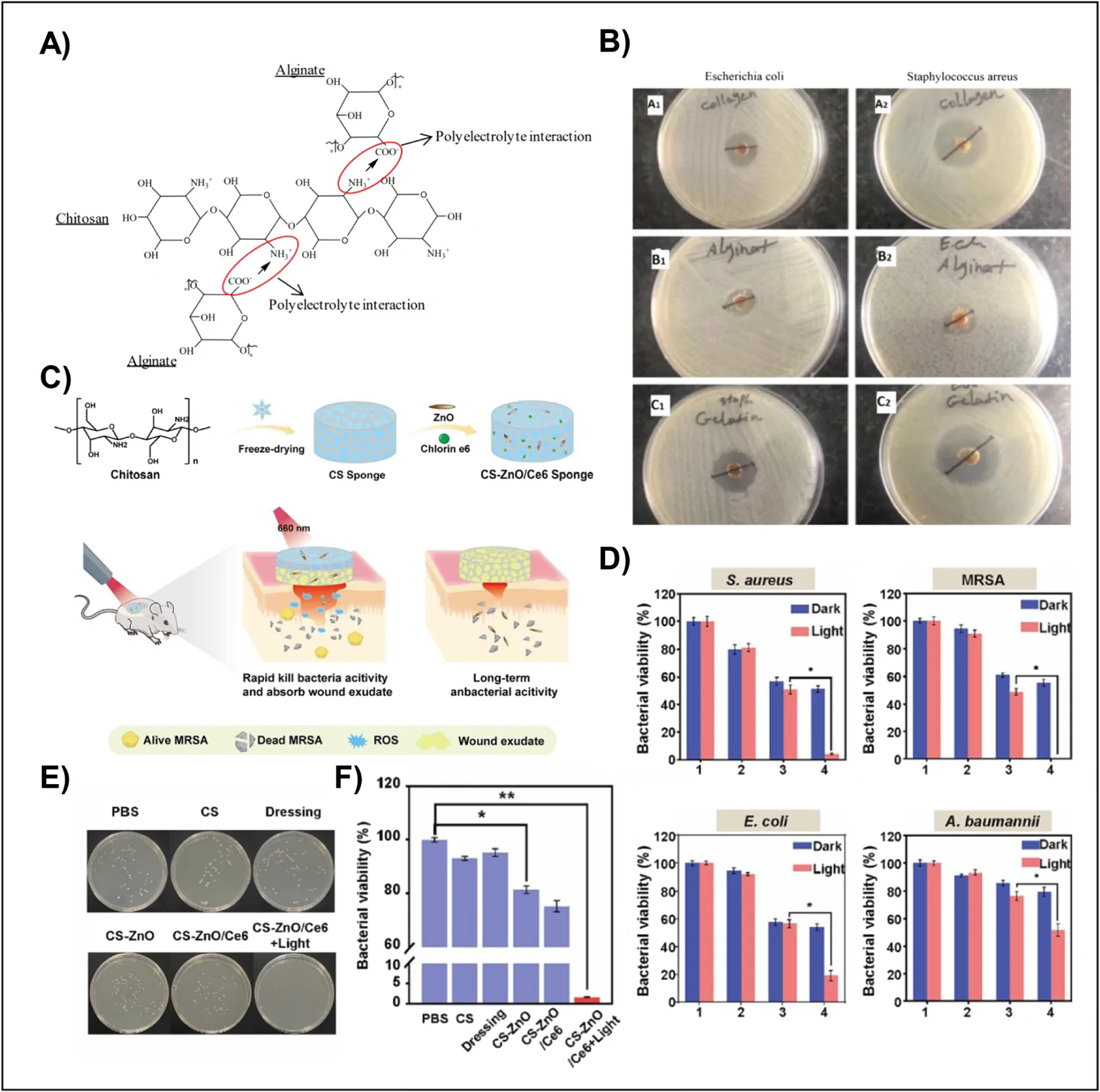
Structure-function relationships in chitosan-based composite sponges. **A)** Chemical structures of chitosan and alginate highlighting polyelectrolyte interactions between chitosan amino groups and alginate carboxyl groups,^51^ reprinted with permission under a Creative Commons CC-BY 3.0 license from [51]. Copyright 2019 IOP Publishing. **B)** Zone-of-inhibition images showing enhanced antibacterial activity against *E. coli* and *S. aureus* for gelatin-chitosan sponges compared to collagen- and alginate-chitosan systems, attributed to differences in curcumin release,^61^ reproduced with permission from Springer Nature, Copyright © 2019. Photodynamic antimicrobial activity and *in vivo* performance of chitosan-based sponges. **C)** Schematic illustration of the photodynamic chitosan sponge and its application in wound healing. **D)** *In vitro* antibacterial activity comparing PBS, chitosan, chitosan-ZnO, and chitosan-ZnO/Ce6 sponges under dark conditions and light irradiation (100 mW cm^–2^, 15 min). **E)** Representative images of bacterial colonies isolated from infected wound tissues following different treatments, including light-activated CS–ZnO/Ce6 (660 nm, 15 min). **F)** Quantitative analysis of bacterial colonies shown in **E)**,^62^ reproduced with permission from Elsevier, Copyright © 2023. [Note: The labels ‘Staphylococcus arreus’ in panel 4B, ‘Acitivity’ in panel 4C, and ‘anbacterial acitivity’ in panel 4C reflect typographical errors in the original published source and refer to ‘Staphylococcus aureus’, ‘Activity’, and ‘antibacterial activity’.]

This improved release profile also translated into superior *in vivo* wound-healing outcomes, including accelerated closure and reduced inflammation compared with other composites and gauze controls. These findings emphasize the importance of matrix–agent interactions, where the choice of the polymer network directly governs drug-release kinetics and antimicrobial efficacy.

Recent advancements in chitosan-based sponges have shifted toward increasingly multifunctional and stimulus-responsive systems, moving beyond passive antimicrobial activity. Strategies such as postfabrication infusion,^63–68^ blending,^24,29,54,59,69–71^ surface coating,^72^ and chemical modification remain widely used to enhance antimicrobial performance and material properties.^17,73–76^ Emerging approaches have focused on advanced, multifunctional designs, including bacteriophage incorporation for selective and nontoxic bacterial targeting,^77^ electrospun layered architectures that mimic skin tissue,^78^ and anisotropic freeze-dried structures that enhance photothermal responses and tissue integration.^79^

In particular, light-responsive antimicrobial systems have attracted significant attention. A chitosan sponge incorporating zinc oxide (ZnO) and chlorin e6 (Ce6) demonstrated effective photodynamic antibacterial activity through ROS generation upon 660 nm light irradiation (Figure 4C).^62^ Following 15 min of light exposure, the composite achieved substantial reductions in bacterial viability, including 96% for *S. aureus*, 98% for MRSA, 80% for *E. coli*, and 50% for *A. baumannii* (Figure 4D). Additionally, the presence of ZnO contributed to prolonged antimicrobial effects, even after irradiation, likely through sustained oxidative stress mechanisms. *In vivo*, this system significantly reduced bacterial burden and promoted wound healing within 2 days while minimizing inflammation (Figure 4E–F). These developments highlight a key evolution in chitosan sponge design: the transition from conventional antimicrobial scaffolds to engineered, multifunctional platforms that integrate controlled drug release, stimulus-responsive activity, and enhanced biological performance, enabling more effective management of infection and tissue regeneration. While the achieved antibacterial activity was sufficient to reduce microbial burden and promote wound healing *in vivo* against the evaluated bacterial strains, further optimization may be required to assess the durability of antimicrobial protection and resistance to bacterial regrowth, which remain critical considerations for open-wound applications.

In contrast to keratin and aloe vera sponges, which are primarily tailored for tissue engineering and infection prevention, GO-based sponges offer a broader range of applications because of their unique electrochemical properties. The incorporation of antimicrobial agents further enhances their efficacy, enabling effective microbial inactivation in both wound-healing and water-treatment contexts. In particular, GO is frequently combined with chitosan to form composite sponges, improving not only the antibacterial performance but also the mechanical strength, cellular compatibility, and hemostatic function.^71,80^ Beyond passive antimicrobial activity, GO-based sponges can readily inactivate microorganisms through electrochemical mechanisms. For example, a graphene sponge modified with boron- and nitrogen-doped reduced graphene oxide (BRGO/NRGO) demonstrated efficient removal of *E. coli* in a continuous flow water treatment system.^81^ Atomic doping, particularly with NRGO, significantly enhanced disinfection performance, achieving up to 5.5-log bacterial reduction with minimal energy input.

Post-treatment analysis using SEM and live/dead staining revealed severe membrane damage and loss of bacterial viability (Figure 5A), consistent with an electroporation-driven mechanism of cell lysis.^82^ Importantly, the low electrocatalytic activity toward chloride oxidation minimizes the formation of harmful disinfection byproducts. This high level of microbial inactivation highlights the strong potential of GO-based sponges for water treatment applications, where substantial bacterial reductions are critical for improving water quality and reducing the risk of waterborne disease transmission. Future studies evaluating the performance against diverse microbial communities and under realistic water conditions could further establish the applicability of these systems for large-scale water purification.

**Figure 5:**
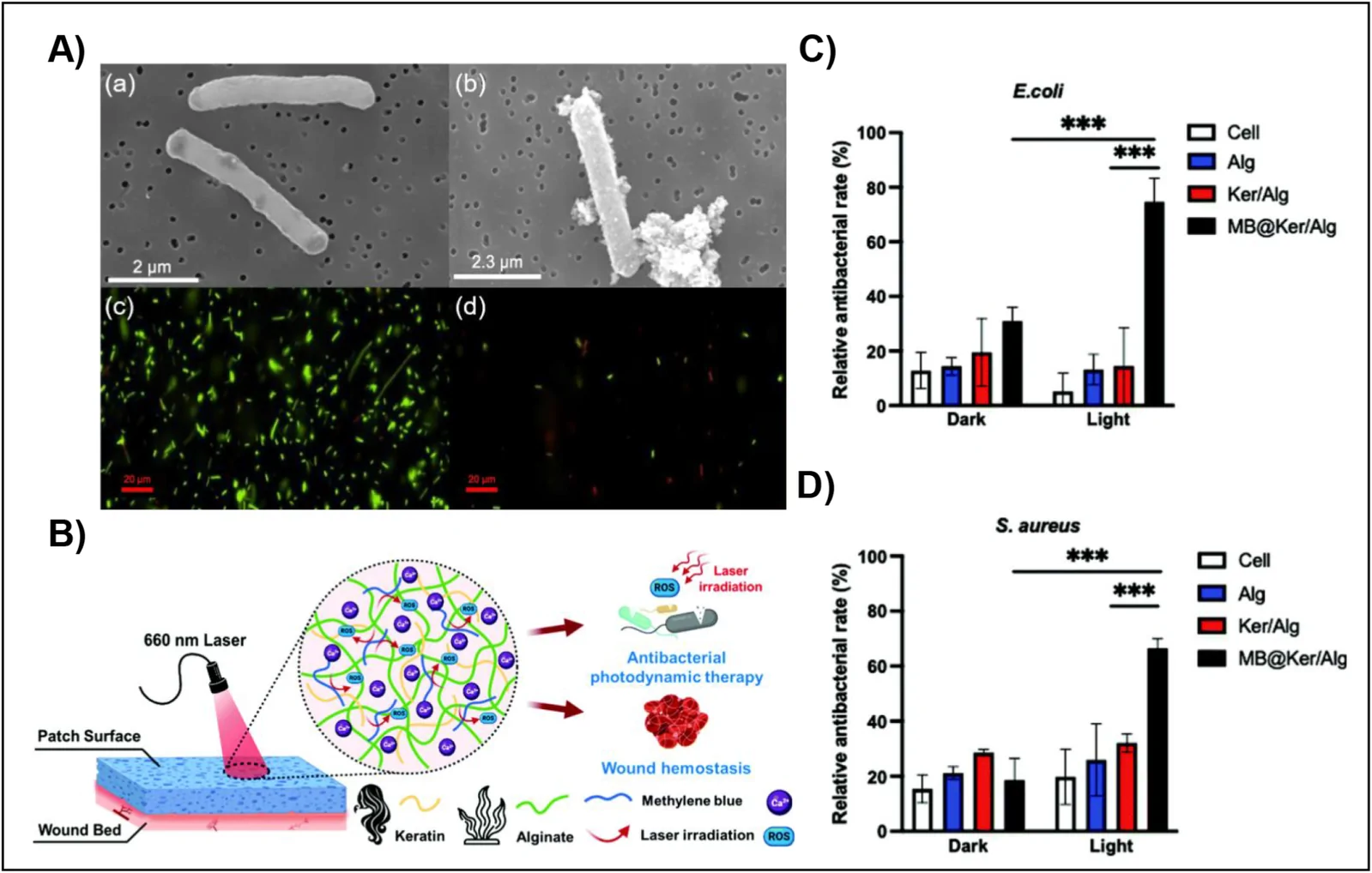
Electrochemical antimicrobial activity of graphene-based sponges for water treatment. **A)** SEM images and Live/Dead staining of *E. coli* before (a,c) and after (b,d) treatment, demonstrating membrane damage and reduced viability,^81^ reproduced with permission from Elsevier, Copyright © 2022. Photodynamic antimicrobial activity of methylene blue-loaded keratin-alginate sponges. **B)** Schematic illustration of methylene blue incorporation into a keratin-alginate sponge to enable light-activated antimicrobial functionality. **C,D)** Antimicrobial activity against planktonic *E. coli* and *S. aureus* with and without 30 min of light irradiation,^83^ reproduced with permission from the Royal Society of Chemistry, Copyright © 2022.

Although the integration of antimicrobial agents into keratin-based sponges remains relatively underexplored, recent studies demonstrate their potential to significantly enhance the antimicrobial performance. Agents including plant-derived compounds and metals,^84^ photosensitizers,^83^ and antiseptics,^85^ have been successfully integrated, resulting in substantial inhibition of both Gram-positive and Gram-negative bacteria. Among these strategies, antimicrobial photodynamic therapy has gained increasing attention because of its ability to generate ROS *in situ*. For instance, methylene blue, a well-known photosensitizer, produces ROS upon activation with visible light, inducing oxidative stress that damages microbial membranes and intracellular components.^86^ Incorporation of methylene blue into a keratin-alginate sponge enabled light-triggered antimicrobial activity (Figure 5B), where irradiation at 660 nm for 30 min resulted in significant reductions in planktonic *E. coli* and *S. aureus* viability by 74.7% and 66.4%, respectively (Figure 5C–D).^83^ While the observed bacterial reductions were moderate, the ability to activate antimicrobial activity only when needed represents a significant advantage for applications requiring localized infection control. Future optimization of photosensitizer loading, light delivery, and treatment duration may further improve antimicrobial efficacy while preserving the spatiotemporal control that distinguishes photodynamic systems from conventional antimicrobial therapies.

Similar to keratin-based systems, antimicrobial sponges derived from aloe vera (AV) remain relatively underexplored.^87^ However, AV has been used to enhance the antimicrobial performance of other polysaccharide-based scaffolds, particularly chitosan.^88^ In one approach, an AV sponge was directly functionalized with amoxicillin and nystatin to provide broad-spectrum antibacterial and antifungal activities for oral applications. The incorporation of these agents significantly improved antimicrobial efficacy against key pathogens associated with dental infections, including *S. mutans*, *A. actinomycetemcomitans*, *E. faecalis*, and *C. albicans*.^48^ Amoxicillin-loaded AV sponges exhibited large inhibition zones ranging from approximately 27 to 29 mm against bacterial strains, exceeding those of control antibiotic disks in some cases, while nystatin-loaded sponges demonstrated enhanced antifungal activity against *C. albicans* (26.0 mm) compared to that of the control (19.3 mm). The unmodified AV sponge also showed inherent antifungal activity, producing a measurable inhibition zone against *C. albicans*. The strong antibacterial and antifungal performance demonstrated against multiple oral pathogens highlights the potential of AV-based sponges as localized drug-delivery platforms for dental and periodontal applications. Future studies evaluating sustained drug release, long-term antimicrobial activity, and performance within complex oral biofilms could further establish their utility for clinical translation.

### 3.3 Functionalization of Noninherently Antimicrobial Sponges

While some sponge materials exhibit intrinsic antimicrobial properties, most synthetic, protein-based, and polysaccharide-derived sponges require the incorporation of antimicrobial agents to effectively inhibit microbial growth. These agents may be introduced during sponge fabrication or incorporated after formation through physical adsorption, chemical conjugation, or encapsulation strategies. The choice of an antimicrobial agent and loading method depends on the intended application, desired release profile, and compatibility with the sponge matrix. Antimicrobial sponges have therefore been developed for diverse applications spanning biomedical uses (wound healing, tissue, and bone engineering), environmental remediation (water treatment and air filtration), food preservation, and dentistry. Representative examples of antimicrobial agents incorporated into sponge materials are summarized in Table 1.

**Table 1:** Examples of Nonantimicrobial Sponge Materials Incorporated with Antimicrobial Agents

Loading strategy	Example agents	Target microbes	Applications	Ref
Covalent functionalization/chemical grafting	Peptides, antibiotics, polymers, plant extracts, quaternary ammonium compounds (QACs)	*E. coli*, *S. aureus*, MRSA, *S. epidermidis*, *P. aeruginosa*	Wound healing, water treatment, flame retardancy, biomedical	^29,89–99^
Blending during fabrication	Metals, nanomaterials, essential oils, antibiotics, peptides, clay minerals, nitric oxide (NO) donors, alkaline nanoparticles, plant extracts, polymers	*E. coli*, *B. cereus*, *S. aureus*, MRSE, MRSA, *B. subtilis*, *P. aeruginosa*, *P. protegens*, *A baumannii*, *E. faecalis*, *S. enterica*, *K. pneumoniae*, *C. albicans*, *S. pyogenes*, *P.* *vulgaris*, *shigella sp*.	Biomedical, energy, environment, water treatment, wound healing, osteomyelitis, prevention, periodontitis	^14^,^56^,^60^,^70^,^100–103,103–146^
Post-fabrication loading (adsorption/infusion)	Polymers, essential oils, peptides, NO donors, antiseptics, metals, antibiotics, antifungals, photosensitizers, plant extracts, bacteriophages, polysaccharides	*Nitzschia*, *E. coli*, *S. aureus*, *S. pyogenes*, *S. epidermidis*, *P. aeruginosa*, *C. albicans*, *B. subtilis*, *A. baumannii*, *K. pneumoniae*	Water treatment, food preservation, wound healing, biomedical, osteomyelitis,	^147–161^
Surface coatings	Metals, phosphorus-based agents, clay minerals, polymers, QACs, plant extracts	*S. aureus*, *E. coli*, *K. pneumoniae*, *P. aeruginosa*, *E. faecalis*	Surface cleaning, flame retardancy, wound healing, water treatment, biomedical, chemotherapy, osteomyelitis	^162–172^
*In situ* polymerization/nanoparticle formation	Metals, photosensitizers, photothermal agents, polymers	*E. coli*, *S. aureus*, *P. aeruginosa*, *B. cereus*	Wound healing, water treatment, air purification, food preservation, biomedical, wearable sensor	^173–186^

The loading strategy significantly influences the antimicrobial release kinetics and long-term activity. Covalent immobilization typically provides sustained antimicrobial activity with minimal leaching, whereas physically adsorbed or infused agents often exhibit an initial burst release, followed by rapid depletion. This effect is particularly pronounced when agents are primarily entrapped within the pore structure, rather than integrated into the polymer matrix. Encapsulation within nanoparticles or polymer carriers can further modulate diffusion-controlled release, enabling prolonged antimicrobial efficacy. These design considerations are particularly important for applications such as wound healing and water treatment, where sustained antimicrobial performance is required.

#### 3.3.1 Synthetic Polymers

Synthetic polymers have become foundational materials in the fabrication of antimicrobial sponges because of their exceptional versatility and tunable physicochemical properties. These materials enable precise control over the pore structure, mechanical strength, and surface chemistry, allowing sponges to be tailored for applications ranging from wound healing to environmental remediation. Their widespread availability, cost-effectiveness, and, in many cases, biocompatibility further support their use in scalable antimicrobial systems.^187,188^ Polymers such as poly­(vinyl alcohol) (PVA), for example, are widely used for their high absorbency and hydrogel-forming capability, making them particularly suitable for fluid management in wound dressings.^100^ A broad range of antimicrobial agents have been incorporated into synthetic polymer-based sponges, including metal-based compounds,^119–123,153,164,165,175–180,182,185^ antibiotics,^124,154^ essential oils,^100,125^ QACs,^93^ antimicrobial polysaccharides,^56,126^ antimicrobial coatings,^94,166,167^ antifouling agents,^168^ NO donors,^150,151^ photosensitizers,^155,174,181^ and plant-derived products.^70,95,111,127–129,162^ However, beyond the choice of the antimicrobial agent, the method of incorporation has emerged as a critical design parameter, influencing agent distribution, release kinetics, and long-term efficacy. In particular, strategies such as incorporation during polymerization (blending), surface coating, adsorption/infusion, and *in situ* nanoparticle growth have been widely explored with distinct trends observed depending on the polymer system.

Polyurethane (PU) sponges represent one of the most widely studied synthetic polymer systems for antimicrobial applications owing to their highly tunable foam chemistry, mechanical resilience, and scalability. While PU is traditionally used in applications such as furniture and insulation,^189^ its porous architecture and ease of fabrication make it an attractive platform for functional modifications. A dominant strategy in recent literature involves postfabrication surface coating, where antimicrobial agents are deposited onto the sponge skeleton to maximize surface exposure and interaction with microorganisms.^94,162^ This approach is particularly advantageous in biomedical applications as it enables the localization of antimicrobial functionality at the material interface while preserving the bulk mechanical properties of the sponge. For example, a multifunctional PU sponge was developed through the asymmetric surface coating of near-infrared (NIR)-responsive superhydrophobic nanoparticles (F-ZnO@Ag) onto a polydopamine-modified PU sponge.^163^ The resulting structure combined a superhydrophobic, antifouling outer layer with an underlying hydrophilic matrix, enabling both the prevention of bacterial adhesion and controlled fluid management at the wound site. The presence of silver nanoparticles provided sustained antimicrobial activity through the release of Ag^+^ ions, while the incorporation of ZnO enabled photothermal conversion under NIR irradiation. As a result, the coated sponge demonstrated broad-spectrum antibacterial activity, achieving approximately 85% bacterial inhibition under ambient conditions and up to 100% bactericidal efficiency against MRSA with NIR irradiation. The enhanced performance is attributed to the synergistic effects of Ag^+^-mediated antimicrobial activity and NIR-induced localized heating, which together disrupt bacterial membranes and accelerate cell death.^190,191^ Beyond antimicrobial efficacy, this coating strategy also enables additional functionalities, including antifouling behavior and unidirectional exudate transport, highlighting the versatility of surface-modified PU sponges for advanced wound-healing applications. These coatings demonstrate how surface engineering can transform conventional PU foams into highly effective platforms for infection control and tissue repair, where the high level of antibacterial efficacy achieved against MRSA suggests a strong potential for managing wound-associated infections in clinical settings.

Polydimethylsiloxane (PDMS), a silicone-based polymer, is widely employed in the fabrication of porous sponges because of its chemical stability, flexibility, and ease of structural tuning. Porous PDMS sponges are typically fabricated using porogen templating methods, such as salt or sugar leaching, which enable precise control over the pore size and overall porosity.^192,193^ The preferred strategy for introducing antimicrobial functionality into PDMS sponges is the direct incorporation or blending of antimicrobial agents into the polymer matrix during fabrication.^130–133^ For example, PDMS sponges were rendered superhydrophobic through the incorporation of graphene nanoplatelets, while copper microparticles were introduced as antimicrobial agents (Figure 6A).^14^

**Figure 6:**
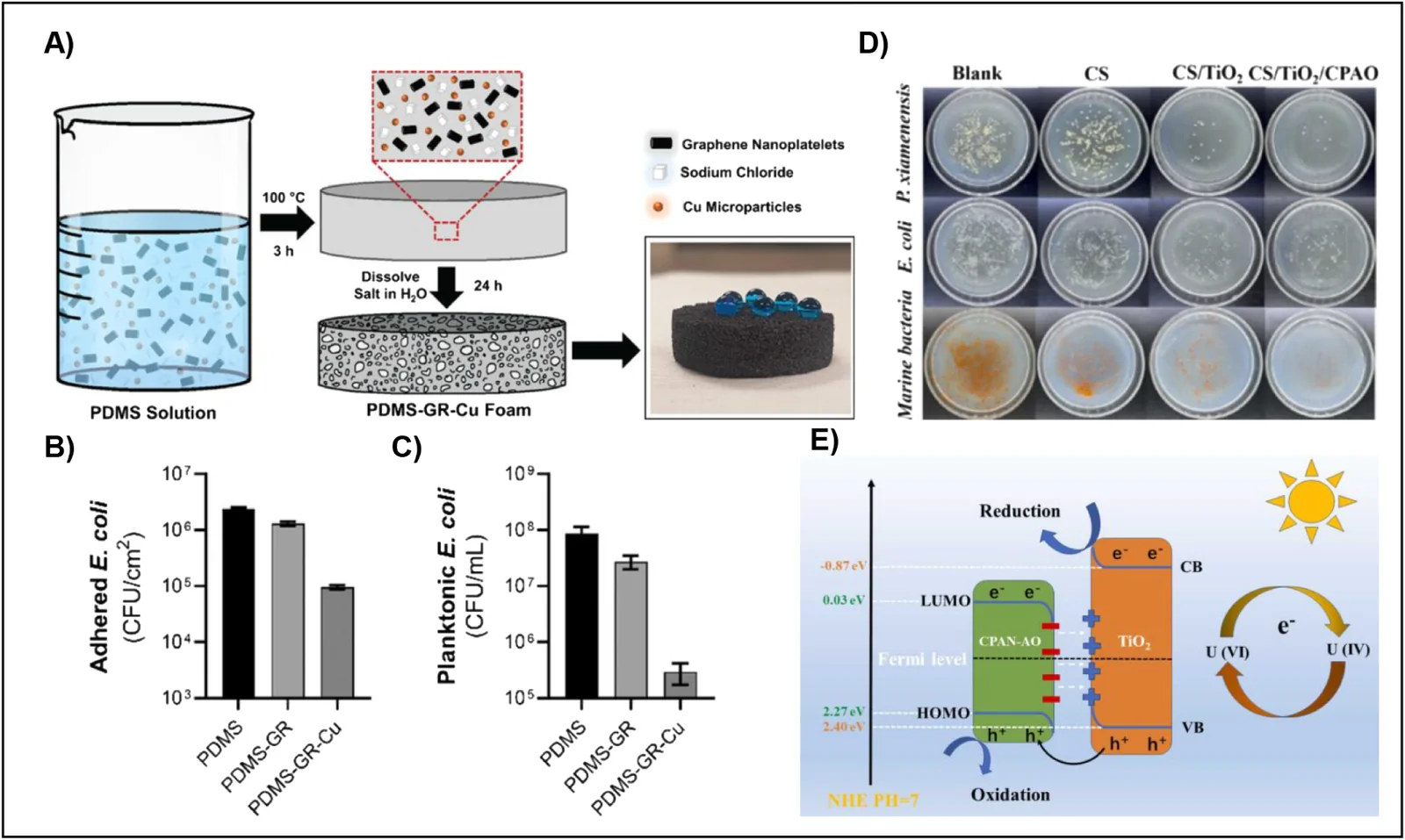
Fabrication and antimicrobial functionality of a graphene nanoplatelet (GR)/copper (Cu)-incorporated PDMS sponge. **A)** Schematic of sponge preparation via a sacrificial salt-templating method, generating an interconnected porous PDMS network after NaCl removal. **B,C)** Antimicrobial performance arises from the combined hydrophobicity and contact-killing effects of GR and Cu, which limit *E. coli* adhesion, along with Cu-induced reactive oxygen species generation and partial agent leaching that reduce planktonic bacterial viability,^14^ reproduced with permission from American Chemical Society, Copyright © 2023. Melamine CS/TiO_2_/CPAN-AO sponge prepared via *in situ* growth of TiO_2_ on the sponge framework, followed by polyacrylonitrile coating and amidoximation to form the functionalized composite. **D)** Antimicrobial activity of TiO_2_ and CPAN-AO, promoting reactive oxygen species generation and reducing microbial adhesion. **E)** Proposed photocatalytic mechanism illustrating light-induced ROS production responsible for microbial inactivation,^194^ reproduced with permission from Elsevier, Copyright © 2023.

This dual-modification strategy combines antifouling and bactericidal mechanisms: the superhydrophobic surface minimizes microbial attachment, while copper induces the generation of reactive oxygen species (ROS), leading to membrane disruption and damage to intracellular components, such as proteins and DNA.^195^

In parallel, graphene-based nanomaterials contribute additional antimicrobial effects through direct interactions with membrane lipids and intracellular biomolecules, ultimately disrupting essential metabolic processes.^196^ As a result of these effects, the modified PDMS sponge achieved high antibacterial performance, with reported killing efficiencies of 99.66% and 96% against planktonic (Figure 6B) and adhered (Figure 6C) *E. coli*. These combined strategies highlight the effectiveness of integrating multifunctional additives within PDMS matrices, enabling efficient oil–water separation, alongside enhanced electrical conductivity for applications in environmental remediation and conductive filtration systems. Further optimization of the conductive network may provide opportunities to enhance interfacial antimicrobial activity through additional mechanisms, such as electrostatic membrane disruption, which could improve performance in water remediation applications, where maintaining water quality and preventing persistent microbial contamination are critical.

In addition to filler-based modifications, PDMS has also been widely explored as a platform for loading antimicrobial agents through solvent-induced swelling and infusion^18,156^ leveraging its inherently permeable polymer network. This approach also allows antimicrobial molecules to diffuse into the polymer matrix and occupy the interconnected pores of the sponge, facilitating a higher loading capacity and uniform distribution. The porous structure of PDMS not only enhances agent retention but also enables tunable release profiles, which are particularly advantageous for short-lived or reactive antimicrobial species. High loading capacities achieved through swelling-based infusion have been increasingly demonstrated with NO donor molecules, which release NO directly from the porous sponge matrix to effectively inactivate bacteria.^150,151^ The interconnected pore network facilitates rapid diffusion and sustained release, allowing for high local concentrations of NO while minimizing systemic exposure. Controlled NO release is critical to balancing antimicrobial efficacy with cytocompatibility, making these systems especially attractive for applications such as infection-resistant medical devices, wound healing, and localized antimicrobial therapies. Importantly, this swelling-based approach enables the incorporation of small molecules and gasotransmitter donors without compromising the bulk mechanical properties of PDMS, preserving its flexibility, compressibility, and high surface area for biological interactions.

Melamine sponges have emerged as a widely used platform for antimicrobial applications due to their highly porous, lightweight structure and ease of surface modification. A widely adopted strategy in these systems is the *in situ* growth or deposition of metal-based antimicrobial agents onto the sponge framework, particularly for water treatment and environmental remediation applications.

Metals such as silver, zinc, copper, iron, and titanium are frequently incorporated because of their well-established antimicrobial properties.^178,180,182,197,198^ Among these, titanium dioxide (TiO_2_) is especially attractive because of its photocatalytic activity, which enables the generation of ROS, including hydrogen peroxide, hydroxyl radicals, and superoxide radicals. These ROS effectively disrupt microbial membranes and intracellular components, leading to cell death.^199^ To further enhance photocatalytic efficiency, TiO_2_ has been conjugated with cyclized ladder-structured conjugated polyacrylonitrile (CPAN), forming a type I heterojunction that improves charge separation and ROS generation.^194^ Through a hydrothermal process, TiO_2_/CPAN-based composites can be grown directly onto carbonized melamine sponge frameworks, producing TiO_2_/CPAN-AO sponges with enhanced antimicrobial and photocatalytic performance. As a result, these sponges exhibit significant reductions in microbial adhesion under simulated seawater conditions due to increased ROS production (Figure 6D) while also enabling simultaneous environmental remediation functions, such as the photocatalytic reduction and removal of uranium species from contaminated water (Figure 6E). This dual functionality highlights the effectiveness of *in situ* growth strategies in melamine sponges, where strong integration between the antimicrobial agent and the sponge substrate enables robust performance in aqueous and environmental systems.

#### 3.3.2 Natural Polymers—Proteins

Proteins stand out as versatile and widely used materials for sponge fabrication because of their unique biological attributes and diverse functionalities. These natural biomacromolecules offer inherent biocompatibility and biodegradability, making them particularly well suited for biomedical applications such as wound healing as well as environmental applications. Their complex structures and intrinsic bioactivity enable interactions with cells and the extracellular matrix, promoting tissue regeneration and healing of open wounds.^200^ Protein-based sponges are typically fabricated through solution-based processing followed by freeze-drying, yielding highly porous and hydrophilic structures that are ideal for the incorporation of antimicrobial agents. A recurring strategy in these systems is the direct blending or incorporation of antimicrobial agents into the protein matrix prior to gelation or lyophilization, allowing for a uniform distribution throughout the scaffold while preserving its structural integrity. This approach not only maintains the inherent wound-healing properties of protein-based materials but can also further enhance them by preventing microbial infection, thereby accelerating the healing process.^201^ A wide range of antimicrobial agents has been incorporated into protein-based sponges using this strategy, particularly in systems derived from silk, collagen, and gelatin, which represent some of the most extensively studied protein matrices. These materials have been functionalized with metals,^134,135,137,173,184^ antimicrobial polymers,^186^ antimicrobial peptides,^96,202^ antimicrobial polysaccharides,^139,141^ NO donors,^161^ and plant derivatives/natural extracts.^117,118,136,138,140,183^ In addition, chemical modifications of protein matrices have been explored to further enhance antimicrobial performance.^98,203^

Silk, a class of fibrous proteins produced by arthropods, has been extensively used in biomaterials because of its biocompatibility, biodegradability, and robust mechanical properties.^204^ Silk is primarily composed of fibroin, a hydrophobic structural protein, and sericin, a hydrophilic adhesive protein that binds fibroin fibers together.^205^ While fibroin is most commonly used for sponge fabrication due to its mechanical strength and stability, the incorporation of sericin has been shown to enhance degradation behavior and cellular interactions, which may be advantageous in specific tissue engineering applications.^206^ In recent literature, silk fibroin sponges have been widely explored as delivery platforms for antimicrobial agents, where their highly porous, freeze-dried structures enable a high loading capacity and controlled release. This is most commonly achieved through blending or postfabrication adsorption of bioactive compounds, mainly including antibiotics, antimicrobial peptides, and natural extracts. These strategies use the interconnected pore network of silk sponges to facilitate sustained antimicrobial activity while maintaining a moist and biocompatible environment that is suitable for wound healing.

Building on this trend, recent studies have increasingly focused on incorporating antimicrobial peptides into silk fibroin sponges as alternatives to traditional antibiotics due to their broad-spectrum activity and reduced risk of resistance development. In one such approach, osteoinductive peptide-enriched silk fibroin (PSF) sponges were functionalized with LL-37-derived peptides (FK-16 and GF-17) through postfabrication loading to achieve efficient incorporation and release.^157^ The sponges exhibited distinct release profiles depending on peptide–matrix interactions, with FK-16 demonstrating rapid and substantial release (>90% within 24 h), which directly correlated with significant bactericidal activity against methicillin-resistant *Staphylococcus epidermidis* strains. In contrast, GF-17 showed a lower release efficiency and limited antimicrobial performance, highlighting the importance of both peptide selection and matrix compatibility in designing effective delivery systems. However, while these results demonstrate the potential of antimicrobial peptide-functionalized sponges as alternatives to conventional antibiotics, achieving comparable clinical antimicrobial performance will require further optimization of sustained bactericidal activity to ensure effective bacterial clearance under more challenging infection conditions.

Similarly, collagen-based systems have gained significant attention, as collagen, an essential extracellular matrix protein, provides a naturally bioactive and cell-interactive platform for tissue repair.^207^ A protein-based multifunctional sponge design was recently demonstrated through acellular dermal matrix (ADM)-derived scaffolds, where collagen-rich matrices were used to provide a biologically active framework for wound healing. In this approach, a porous sponge was fabricated via freeze-drying and subsequently functionalized through the blended incorporation of curcumin-loaded mesoporous cerium oxide nanoparticles (CeO_2_-cur), enabling a uniform distribution throughout the sponge.^183^ The resulting composite sponge exhibited strong, broad-spectrum antibacterial activity, achieving 83.97% and 87.63% bacterial reductions against *E. coli* and *S. aureus*, respectively (Figure 7A,B,G,I). Live/dead staining further revealed extensive bacterial death following treatment, with a marked shift from green (viable) to red fluorescence (nonviable) (Figure 7C,D,H,J). Morphological analysis via scanning electron microscopy showed intact, smooth bacterial surfaces in control groups, whereas treated bacteria exhibited severe membrane disruption, including wrinkling, collapse, and leakage of intracellular contents (Figure 7E-F).

**Figure 7:**
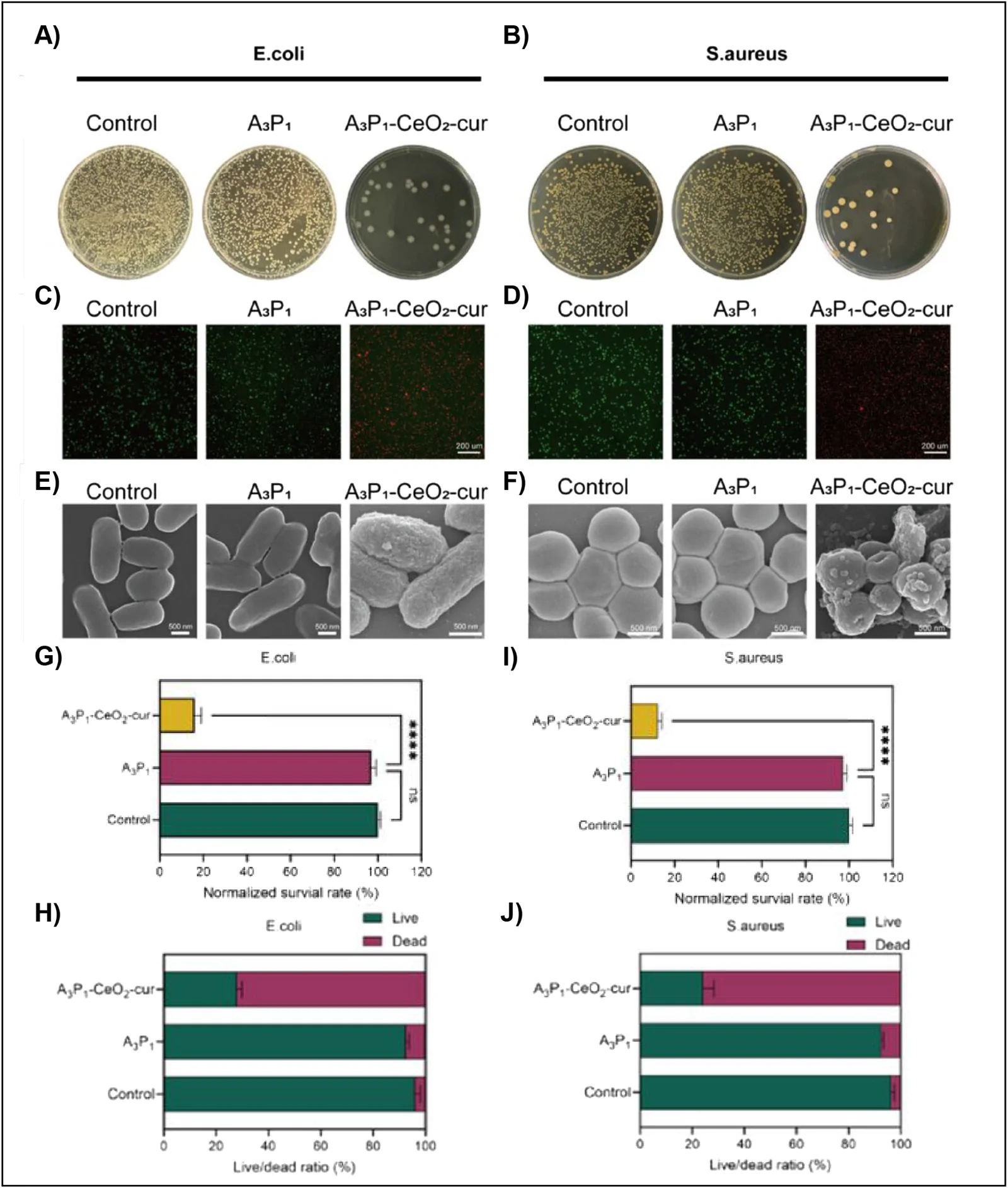
In vitro antibacterial performance of the A3P1-CeO_2_-cur collagen-based sponge scaffold. **A,B)** Representative agar plate images showing *E. coli* and *S. aureus* colonies following co-incubation with different scaffold formulations. **C,D)** Live/dead fluorescence staining images (green: viable cells; red: non-viable cells). **E,F)** Scanning electron microscopy images illustrating bacterial morphological changes after treatment. **G,H)** Quantitative analysis of bacterial survival rates. **I,J)** Corresponding live/dead bacterial ratios,^183^ reproduced with permission from John Wiley & Sons, Copyright © 2025. [Note: The labels ‘Normalized survial rate’ in panels 7G,I reflect typographical errors in the original published source and refer to ‘Normalized survival rate’].

Mechanistically, this antibacterial performance was attributed to a synergistic effect: curcumin disrupts bacterial membrane integrity through insertion into lipid bilayers,^208^ while ceria nanoparticles promote localized ROS generation,^209^ collectively leading to membrane damage and bacterial death. Beyond antimicrobial activity, the collagen-based scaffold provided intrinsic biological functionality, supporting cell adhesion, migration, and modulation of the inflammatory response. The material promoted macrophage polarization toward a prohealing phenotype, while its high swelling capacity enabled effective exudate management, both of which are critical factors in chronic wound environments such as diabetic ulcers.

Although the bacterial reductions achieved by this material are lower than those reported for some highly potent antimicrobial systems, the observed decrease in the microbial burden was sufficient to support improved wound-healing outcomes *in vivo*. This highlights that antimicrobial performance requirements are highly dependent on the application, where reducing the bacterial load while promoting tissue regeneration may be more clinically relevant than achieving complete bacterial eradication.

Overall, these studies highlight a broader trend in protein-based antimicrobial sponges, where biologically derived matrices are preferentially combined with natural or bioinspired antimicrobial agents, such as peptides and plant-derived compounds, to maximize biocompatibility while maintaining therapeutic efficacy. Compared with synthetic polymers, protein-based materials such as silk, collagen, and gelatin inherently support cell–matrix interactions, making them particularly advantageous for biomedical applications requiring both antimicrobial action and tissue regeneration. In addition to emerging alternatives such as antimicrobial peptides, conventional antibiotics continue to be incorporated into protein-based sponges, not as standalone therapies but as controlled delivery systems. By localizing antibiotic release within the porous scaffold, these systems can enhance therapeutic efficiency, reduce systemic exposure, and help mitigate the development of antimicrobial resistance.^101,110,207,210^ Together, these strategies highlight the evolution of protein-based sponges from passive wound dressings to active multifunctional platforms capable of integrating antimicrobial, immunomodulatory, and regenerative functions.

#### 3.3.3 Natural Polymers—Polysaccharides

Similar to proteins, polysaccharides have emerged as prominent candidates for the fabrication of sponges. These complex carbohydrates, composed of long chains of sugar units, are naturally derived from sources such as algae, plants, and microorganisms.^211^ Their inherent biological roles, ranging from immune recognition to cell signaling, ensure safe interactions with biological systems, making them particularly attractive for biomedical applications.^212^ Polysaccharides can be broadly categorized based on their charge. Chitosan, previously discussed as an intrinsically antimicrobial polysaccharide, is a notable example of a cationic polysaccharide. Anionic polysaccharides, including alginate, pectin, gellan gum, and ricolin, carry negatively charged functional groups that enable interactions with ions, biomolecules, and cells, supporting applications in tissue regeneration and drug delivery.^213,214^ In contrast, neutral polysaccharides such as cellulose, dextran, agarose, starch, and levan primarily contribute to structural integrity and mechanical stability.^214,215^ Unlike proteins and nucleic acids, polysaccharides often form branched architectures through modification of functional groups along their backbones, allowing for extensive tunability in both physical and biological properties.^216^ When processed into porous sponges, polysaccharides provide highly hydrated, biocompatible matrices that can be readily functionalized with antimicrobial agents. As a result, polysaccharide-based antimicrobial sponges have been explored for tissue engineering, wound healing, food preservation, water disinfection, and oral health applications. To impart antimicrobial functionality, these materials have incorporated a wide range of agents, including metals,^103,103,109,112,143–146,172^ essential oils,^104^ photosensitizers,^217^ clay/inorganic minerals,^105,142^ antimicrobial peptides/polymers,^149,218^ polysaccharides,^57,219^ organosulfurs,^220^ bacteriophages,^159^ antibiotics,^23,113,116^ plant derivatives,^60,89,115,158,221,222^ and insect extracts.^223^ Chemical modification of polysaccharides may also introduce antimicrobial properties.^97,99,102,148,160^

Cellulose, one of the most abundant biopolymers derived from plants, algae, and microorganisms, has been widely explored for antimicrobial sponge fabrication because of its low toxicity, biodegradability, and robust mechanical properties.^224,225^ Its abundant hydroxyl groups enable hydrogen bonding as well as facile chemical functionalization, making cellulose an ideal platform for antimicrobial modification.^226^ Cellulose-based sponges have been extensively explored for applications in food packaging and preservation, where preventing microbial contamination and physical degradation is critical. In this context, antimicrobial functionalization strategies are designed not only to inhibit microbial growth but also to extend the shelf life and maintain product quality. For example, cellulose sponges incorporating antimicrobial agents have demonstrated strong efficacy, including over 99% inhibition of *Rhizopus stolonifer*, a common spoilage fungus, thereby significantly reducing fungal contamination and associated physical damage to perishable foods.^227^

Chemical modification of cellulose has emerged as a common strategy for imparting antimicrobial functionality. For instance, cellulose can undergo oxidation to introduce carboxyl groups on the sponge surface, suppressing the biofilm-synthesis factor cyclic di-GMP, preventing microbial adhesion, and reducing cell membrane permeability.^228^ Oxidized cellulose sponges have been shown to inhibit approximately 92.4% of *S. aureus* and 85.2% of *E. coli*, while also enhancing the preservation of chilled meat.^229^

To further enhance the antimicrobial performance, oxidized bacterial cellulose sponges were functionalized with cinnamon essential oil (CEO)-loaded microcapsules via electrostatic adsorption.^148^ The incorporation of CEO significantly increased antibacterial activity, with inhibition zones expanding from 9.2 and 8.6 cm (without CEO) to 23.7 and 16.2 cm against *S. aureus* and *E. coli*, respectively (Figure 8A). This enhanced activity is attributed to bioactive compounds, such as cinnamaldehyde and eugenol, which disrupt microbial membranes and interfere with enzymatic functions.^230^ Beyond direct contact-killing, the system also demonstrated vapor-phase antimicrobial activity. In a gas-phase assay, the functionalized sponges were positioned on Petri dish lids to enable noncontact exposure to bacterial cultures. After 24 h, microbial growth was inhibited by approximately 65% for *E. coli* and 75% for *S. aureus* (Figure 8B–C), whereas control sponges without CEO showed negligible effects. The ability of volatile antimicrobial compounds to diffuse from the sponge matrix enables sustained, noncontact inhibition. Together, these results highlight a broader trend in polysaccharide-based sponges toward multifunctional systems that combine chemical modification with bioactive agent incorporation, enabling both contact-based and vapor-phase antimicrobial activities for applications such as food preservation.

**Figure 8:**
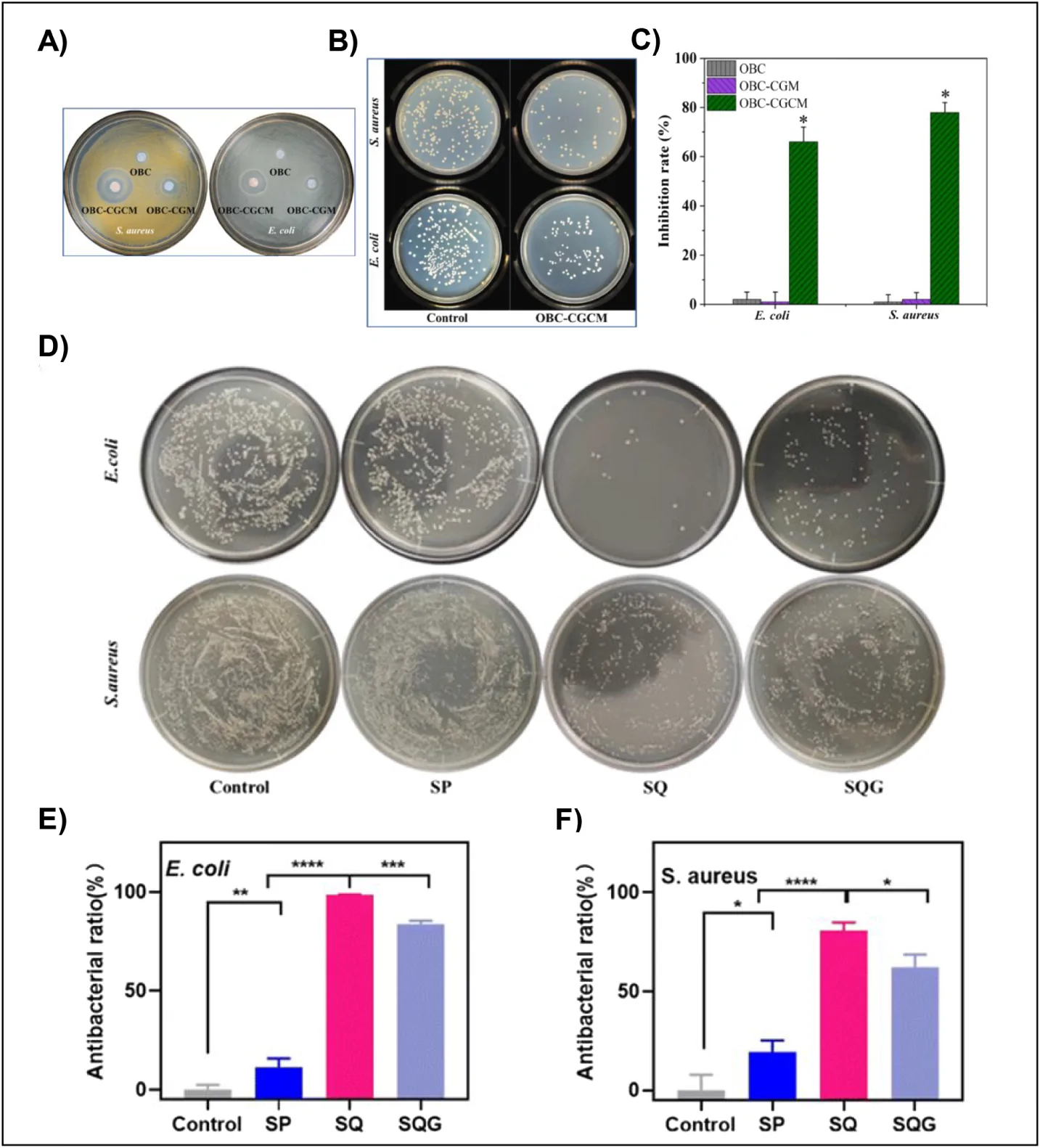
Fabrication and antimicrobial performance of oxidized bacterial cellulose-based sponges. **A)** Representative zone-of-inhibition images against *S. aureus* and *E. coli*. **B)** Qualitative and **C)** quantitative evaluation of antimicrobial activity against *S. aureus* and *E. coli*,^148^ reproduced with permission from Elsevier, Copyright © 2024. *In vitro* antibacterial activity of shape-memory starch sponges with quaternary ammonium starch (QSt), enhancing surface accessibility and antibacterial interaction with bacterial membranes. **D)** Representative images of *E. coli* and *S. aureus* cultures after incubation with different sponge formulations for 12 h. Quantitative antibacterial ratios against **E)** *E. coli* and **F)** *S. aureus*,^160^ reproduced with permission from the Royal Society of Chemistry, Copyright © 2023.

Although the near-complete inhibition of *R. stolonifer* represents strong antimicrobial performance, lower levels of microbial suppression, such as those achieved with CEO-functionalized sponges, may still provide meaningful benefits for food preservation by slowing spoilage progression and extending the shelf life. Importantly, the antimicrobial performance should also be considered in the context of the target microorganism, as different food-associated microbes may exhibit varying susceptibility to antimicrobial strategies. These differences highlight that antimicrobial efficacy must be interpreted alongside the intended application and microbial target, where controlling spoilage fungi or bacterial contamination may require different levels of activity.

In the context of wound healing, essential oils, particularly cannabidiol (CBD) oil, have been successfully employed.^231^ A levan sponge containing 20 wt % CBD oil significantly inhibited *S. aureus* and *P. aeruginosa* growth, maintaining maximum inhibitory effects even after 3 months of storage.^104^ Levan, a fructan biopolymer, holds promise in the food industry as well, as it can be used in edible forms. The antimicrobial potential of levan biomaterials is further enhanced when essential oils are incorporated, due to the slower and prolonged leaching of the oils from the sponge matrix, allowing effective microbial inhibition with smaller quantities.^232^ This oil not only damages microbial cell envelopes but also provides antioxidant, anti-inflammatory, and protein adsorption-enhancing effects, supporting healthy wound healing by preventing infection and promoting essential processes such as blood coagulation.

While naturally derived antimicrobial agents such as essential oils offer advantages including biocompatibility and multifunctionality, chemical modification of polysaccharide-based sponges remains a widely employed strategy to impart more robust and tunable antibacterial properties. In particular, the incorporation of cationic functionalities enables strong electrostatic interactions with negatively charged bacterial membranes, leading to membrane disruption and cell death.^233^ For example, a starch-based sponge system was developed by crosslinking carboxylated starch, followed by post-loading with quaternary ammonium starch (QSt) and gelatin.^160^ Rather than modifying the bulk starch network directly, this post-loading approach allows the cationic QSt to remain more accessible at the sponge interface, enhancing its interaction with bacterial cell membranes. As a result, the QSt-loaded sponge exhibited significantly improved antibacterial activity (Figure 8D), achieving inhibition ratios of 98.4% against *E. coli* (Figure 8E) and 80.5% against *S. aureus* (Figure 8F), compared to minimal activity in the unmodified scaffold. This antibacterial effect is primarily attributed to electrostatic disruption, where the positively charged quaternary ammonium groups interact with negatively charged phospholipids in the bacterial membrane, compromising membrane integrity. Although the addition of gelatin slightly reduced antibacterial efficacy, it improved biocompatibility and mechanical stability, maintaining favorable properties for wound healing, including a porous structure for fluid absorption, good cell compatibility, and effective hemostatic performance. While the QSt-loaded sponge demonstrated strong inhibition of *E. coli*, the lower reduction observed against *S. aureus* highlights the importance of evaluating antimicrobial performance across diverse bacterial species with varying susceptibility. Although the achieved reductions, combined with the scaffold’s favorable wound healing properties, suggest potential for reducing bacterial burden in wound environments, further improvements in antimicrobial potency and evaluation against additional clinically relevant pathogens, including biofilm-forming strains, could strengthen the translational potential of these systems.

## 4 Conclusions and Future Directions

Antimicrobial sponges have emerged as versatile materials for applications ranging from wound dressings and biomedical devices to environmental remediation and water purification. Across these diverse applications, several recurring design principles have been identified, reflecting how material selection, structural engineering, and antimicrobial strategies are tailored to specific functional demands (Table 2).

**Table 2:** Major Design Trends in Antimicrobial Sponge Materials across Different Applications

Application	Polymers	Antimicrobial agents	Tested microbes	Design focus
Wound healing	Cellulose, chitosan, gelatin, alginate, collagen	Metals, natural extracts, photosensitizers, chemical modification	*S. aureus*, MRSA, *E. coli*, *P. aeruginosa*, *C. albicans*	Biocompatible, hemostatic, cell proliferative, controlled release
Water treatment/filtration	Melamine, graphene oxide, PDMS	Metals, chemical modification	*E. coli*, *P. aeruginosa*	Reusability, rapid inactivation, filtration efficiency
Food preservation/packaging	Cellulose, starch, chitosan, alginate	Essential oils, bacteriocins, natural extracts	*S. aureus*, *E. coli*, *S. enterica*	Sustained/vapor-phase release, food safety, shelf-life extension
Environmental remediation (air/surface)	Melamine, PU, chitosan	Metals, photosensitizers, natural extracts	*E. coli*, *S. aureus*	Self-disinfection, continuous activity, stimuli-responsiveness

One of the most important considerations in antimicrobial sponge development is the choice of the polymer matrix. Both natural and synthetic polymers have been widely used, with each offering distinct advantages. Natural polymers such as chitosan, cellulose, and collagen are frequently employed due to their biocompatibility, biodegradability, and intrinsic bioactivity, with certain materials, such as chitosan, also exhibiting inherent antimicrobial properties. In contrast, synthetic polymers including PU, PVA, and PDMS provide greater mechanical stability, chemical resistance, and tunable physical properties, making them well suited for engineering-oriented applications such as filtration systems and durable antimicrobial surfaces. In many cases, hybrid systems combining natural and synthetic polymers are employed to balance biological functionality with structural robustness.

Fabrication techniques also play a critical role in defining the structural and functional properties of the antimicrobial sponges. Common methods, such as freeze-drying, porogen templating, gas foaming, and electrospinning, are widely used to generate highly porous architectures with interconnected pore networks. Among these, freeze-drying remains one of the most frequently used approaches due to its simplicity and ability to produce highly porous scaffolds that are particularly well suited for drug delivery and wound-healing applications.

The incorporation of antimicrobial agents represents a key design parameter. These agents can be integrated into sponge matrices through several strategies, including blending during fabrication, postfabrication adsorption or infusion, covalent grafting, surface coatings, and *in situ* nanoparticle formation. Blending is widely used due to its simplicity and compatibility with conventional fabrication methods, whereas covalent immobilization enhances the stability and reduces the leaching of active agents. Postfabrication infusion offers flexibility in loading but may result in faster release kinetics. Alternatively, surface coatings and *in situ* nanoparticle formation can localize antimicrobial activity at the material interface while minimizing the total amount of active agent required.

A recurring theme in antimicrobial sponge design is that optimal performance is highly application-dependent. Biomedical applications such as wound dressings prioritize biocompatibility, controlled antimicrobial release, and tissue integration, whereas applications such as water purification emphasize mechanical durability, long-term reusability, and broad-spectrum antimicrobial activity. As a result, the selection of polymer systems, antimicrobial agents, and incorporation strategies varies significantly depending on the intended use.

Across these applications, several overarching trends have been observed. Metal-based antimicrobial systems, particularly those involving silver and other metal nanoparticles, are widely used because of their strong and broad-spectrum antimicrobial activity. These systems are especially prevalent in synthetic polymer-based sponges and in applications such as water treatment, where cytotoxicity is less of a concern. In contrast, their use in biomedical contexts, particularly wound healing, is often more carefully controlled because of potential cytotoxic effects on surrounding cells. Natural polymer-based sponges, especially those derived from proteins and polysaccharides, are more commonly used in wound-healing applications due to their favorable biocompatibility and ability to support cellular attachment and tissue regeneration. Among these, chitosan-based systems have received significant attention owing to their intrinsic antimicrobial properties and versatility in combination with other polymers and antimicrobial agents.

Emerging antimicrobial strategies have further expanded this landscape. Nitric oxide-releasing systems and other gasotransmitter-based platforms are gaining interest for their multifaceted antimicrobial, proangiogenic, and immunomodulatory properties, making them particularly attractive for biomedical and wound-healing applications. In parallel, natural antimicrobial agents derived from plant and animal sources offer biocompatible alternatives, often used in combination with natural polymer matrices to enhance the antimicrobial performance while maintaining safety.

Despite significant advances, several challenges remain in the development of antimicrobial sponge materials. Key limitations include achieving sustained antimicrobial efficacy without an undesirable burst release or depletion of active agents, maintaining structural and mechanical integrity under operational conditions, and ensuring the safety, stability, and environmental compatibility of incorporated antimicrobial components. Importantly, many antimicrobial strategies also involve material-specific trade-offs. Metal-based antimicrobials, while highly effective against a broad spectrum of microorganisms, may induce cytotoxicity and oxidative stress toward mammalian cells at elevated concentrations and can accumulate in the environment.^234^ Essential oils and other natural products often exhibit limited stability, volatility, and batch-to-batch compositional variability, which can affect their long-term efficacy.^148^ Antibiotic-loaded sponges remain susceptible to the emergence of antimicrobial resistance and may lose effectiveness against multidrug-resistant pathogens.^39^ Similarly, photosensitizer-based systems require external light activation, limiting their use in deep or inaccessible tissues, while bacteriophage-based approaches generally possess narrow host specificity and require careful matching to the target pathogen.^181^ While strategies such as chemical functionalization and hybrid polymer systems have improved control over release behavior and mechanical performance, they also introduce additional considerations related to complexity, reproducibility, and long-term functionality. Moving forward, future research should focus on multifunctional sponge systems that integrate controlled antimicrobial delivery with enhanced structural design, adaptive or stimulus-responsive behavior, and emerging antimicrobial modalities, such as gasotransmitter-releasing and biologically targeted systems.

Overall, antimicrobial sponges represent a rapidly evolving class of materials with significant potential across biomedical, environmental, and industrial applications. Continued advances in polymer selection, fabrication strategies, and antimicrobial integration will further expand their functionality and enable more effective and application-specific designs.
